# Prolonged dual antiplatelet therapy in patients with non‐ST‐segment elevation myocardial infarction: 2‐year findings from EPICOR Asia

**DOI:** 10.1002/clc.23322

**Published:** 2020-01-22

**Authors:** Yanan Zou, Shuang Yang, Shipeng Wang, Bo Lv, Lili Xiu, Lulu Li, Stephen W.‐L. Lee, Chee Tang Chin, Stuart J. Pocock, Yong Huo, Bo Yu

**Affiliations:** ^1^ Department of Cardiology The Second Affiliated Hospital of Harbin Medical University Harbin Heilongjiang China; ^2^ Department of Medicine Queen Mary Hospital Hong Kong SAR China; ^3^ Department of Cardiology National Heart Centre Singapore Singapore; ^4^ Department of Medical Statistics London School of Hygiene and Tropical Medicine London UK; ^5^ Peking University First Hospital Beijing China

**Keywords:** acute coronary syndrome, antithrombotic management pattern, dual antiplatelet therapy, EPICOR Asia, NSTEMI

## Abstract

**Background:**

Patients with non‐ST‐segment elevation myocardial infarction (NSTEMI) have a generally poor prognosis and antithrombotic management patterns (AMPs) used post‐acute coronary syndrome (ACS) remain unclear. Duration of dual antiplatelet therapy (DAPT) and patient characteristics was evaluated in NSTEMI patients enrolled in EPICOR Asia.

**Hypothesis:**

Patients stopping DAPT early may benefit from more intensive monitoring.

**Methods:**

EPICOR Asia was a prospective, real‐world, primary data collection, cohort study in adults with an ACS, conducted in eight countries/regions in Asia, with 2 year follow‐up. Eligible patients were hospitalized within 48 hours of symptom onset and survived to discharge. We describe AMPs and baseline characteristics in NSTEMI patients surviving ≥12 months with DAPT duration ≤12 and > 12 months post‐discharge. Clinical outcomes (composite of death, myocardial infarction, and stroke; and bleeding) were also explored.

**Results:**

At discharge, 90.8% of patients were on DAPT (including clopidogrel, 99%). At 1‐ and 2‐year follow‐up, this was 79.2% and 60.0%. Patients who stopped DAPT ≤12 months post‐discharge tended to be older, female, less obese, have prior cardiovascular disease, and have renal dysfunction. While causality cannot be inferred, the incidence of the composite endpoint over the subsequent 12 months was 10.6% and 3.1% with shorter vs longer use of DAPT, and mortality risk over the same period was 8.4% and 1.6%.

**Conclusions:**

Over 90% of NSTEMI patients were discharged on DAPT, with 60% on DAPT at 2 years. Patients stopping DAPT early were more likely to have higher baseline risk and may therefore benefit from more intensive monitoring during long‐term follow‐up.

## 1 INTRODUCTION

Despite advances in medical and invasive management strategies for patients with coronary heart disease (CHD), recent results from the Global Burden of Disease Study showed that CHD remained the leading cause of premature death worldwide in 2016, with an age‐standardized mortality rate of 149.7 per 100 000.^1^ While mortality from CHD has progressively declined since 1990 in middle‐ and high‐income countries, it has increased in low‐income regions.^1^ Suggested causes include the growing proportion of people aged ≥60 years, lifestyle factors (such as tobacco and alcohol use, and increasing consumption of foods with high energy/fat content), lack of preventative measures, low availability of CHD management, and lack of rehabilitation and secondary prevention programmes.^2^, ^3^ In fact, increasingly westernized lifestyles have been implicated in the increasing prevalence of overweight, diabetes, and associated cardiovascular diseases (CVD) among affluent people within low‐ to middle‐income countries.^3^

In the United States, approximately 70% of patients presenting with an acute coronary syndrome (ACS) have a non‐ST‐segment elevation (NSTE)‐ACS, comprising non‐ST‐segment elevation myocardial infarction (NSTEMI) and unstable angina (UA), and only 30% have an ST‐segment elevation myocardial infarction (STEMI).^4^ In Asia, the ratio is reversed, with 60‐80% of patients presenting with STEMI, but there is evidence that the proportion of Asian patients with NSTE‐ACS is growing.^5^ Furthermore, although short‐term mortality is higher with STEMI, the risk of death during long‐term follow‐up is greater with NSTEMI.^6^, ^7^

Recent guidelines in the United States and Europe include a class 1 recommendation for 12 months of dual antiplatelet therapy (DAPT) with aspirin and a P2Y_12_ inhibitor following ACS.^4^, ^8^, ^9^ However, in some populations with high risk, 12 months' duration of DAPT is not long enough. The subgroup analysis of the Clopidogrel for High Atherothrombotic Risk and Ischemic Stabilization, Management, and Avoidance trial showed that patients with clinically evident atherothrombosis (documented prior MI, ischemic stroke, or symptomatic peripheral artery disease) benefitted from long‐term DAPT (median duration 27.6 months) beyond aspirin alone.^10^, ^11^ Another trial, PEGASUS‐TIMI 54 (Prior Heart Attack Using Ticagrelor Compared to Placebo on a Background of Aspirin‐Thrombolysis in Myocardial Infarction 54) showed that in patients with a MI more than 1 year previously, treatment with long‐term DAPT (median duration 33 months) significantly reduced the risk of cardiovascular (CV) death, MI, or stroke compared with aspirin therapy alone.^12^ Several meta‐analyses and a systematic review of randomized trials have also indicated that prolonging DAPT to more than 1 year following myocardial infarction (MI) can reduce CV event rates, although at the risk of increased (nonfatal) bleeding.^13^, ^14^, ^15^, ^16^ However, the benefits of continuing DAPT beyond a year in real‐world populations are less well defined, and there is relatively little evidence from Asian countries. The EPICOR Asia (long‐tErm follow‐uP of antithrombotic management patterns In Acute CORonary syndrome patients in Asia) study was an observational, primary data collection study designed to investigate acute and long‐term antithrombotic management patterns (AMPs) in ACS survivors. The aim of this analysis was to evaluate long‐term use of DAPT, and baseline patient characteristics associated with earlier DAPT discontinuation vs >12 months of DAPT, in patients with NSTEMI.

## 2 METHODS

### 2.1 Study design and patients

EPICOR Asia (NCT01361386; https://clinicaltrials.gov/ct2/show/NCT01361386?term=01361386&rank=1) was a prospective, real‐world, cohort study with a 2 year follow‐up period in patients surviving the initial hospitalization for an ACS. Full details of the study design have been published previously.^17^ Briefly, between June 2011 and May 2012, patients were enrolled at discharge from 218 centers in China, Hong Kong, India, Malaysia, Singapore, South Korea, Thailand, and Vietnam. Male and female patients were eligible for inclusion if they were aged ≥18 years, were hospitalized within 48 hours of symptom onset, survived to discharge, and had a final diagnosis of STEMI, NSTEMI, or UA. Patients with a secondary ACS (ie, precipitated by or occurring as a complication of another event, such as surgery, trauma, or percutaneous coronary intervention [PCI]) were excluded from the study. Baseline and in‐hospital data were collected retrospectively for the index event up to hospital discharge, and prospectively by means of telephone interviews at 6 weeks then every 3 months up to 2 years.

All patients provided written informed consent to participate in the study and agreed to follow‐up interviews. The study was conducted in accordance with ethical principles that are consistent with the Declaration of Helsinki revision, International Conference on Harmonization Good Clinical Practice guidelines, and the applicable legislation on non‐interventional studies, and written approval was obtained from the relevant local Ethics Committees and/or Regulatory Authorities.

### 2.2 Objectives

The primary objective of EPICOR Asia was to describe acute and long‐term AMPs in ACS survivors across a range of clinical settings in the eight different countries/regions in Asia. The objectives of the present post hoc analysis were to evaluate reported use of AMPs at discharge and during follow‐up in the sub‐population of patients with NSTEMI and to investigate possible differences in baseline characteristics according to DAPT duration ≤12 months vs >12 months up to the final follow‐up at 2 years in patients discharged on DAPT who survived ≥12 months post‐discharge. The incidence of major adverse CV events (composite of death, MI, and ischemic stroke; and death as an individual outcome) and major bleeding events during the second year post‐discharge by DAPT duration ≤12 vs >12 months is also described. In practice, final interviews were conducted within ±2 weeks of the 24‐month time point. Outcomes data are provided based on cumulative summary at pre‐defined intervals during follow‐up.

### 2.3 Statistical analysis

Baseline demographics and characteristics were compared according to post‐discharge DAPT duration ≤12 months vs >12 months up to 2 years among NSTEMI patients discharged on DAPT who survived ≥12 months post‐discharge. *P* values were calculated using the chi‐square test for categorical variables, two‐sample *t*‐test for comparison of means, and Wilcoxon test for comparison of medians. Patients on DAPT at discharge include all those discharged on two or more antiplatelets (ie, including triple antiplatelet therapy) from the index hospitalization, and DAPT duration is defined as the time from discharge to the last use of two or more antiplatelets, not accounting for interruptions.

In NSTEMI patients who survived at least the first 12 months post‐discharge, the incidence of major adverse cardiovascular events, death as an individual item, and major bleeding events during the second year of follow‐up was also explored according to DAPT duration ≤12 months vs >12 months. A major caveat is that causality cannot be inferred between outcomes and DAPT duration since it was not a randomized study; DAPT duration is not a baseline variable but an outcome, and there are multiple possible confounders, particularly regarding the nonspecified selection processes determining which patients stop or continue on DAPT at any time point. Thus, patients with DAPT duration ≤12 or > 12 months should not be considered treatment groups, but rather as different patient groups with different clinical characteristics and risk profiles.

## 3 RESULTS

### 3.1 Patients

Of the 13  005 ACS survivors enrolled in EPICOR Asia, 12  922 met the inclusion/exclusion criteria, of whom 6306 (48.8%) had a final diagnosis of NSTE‐ACS at discharge, including 2549 (19.7%) with NSTEMI. The mean age of NSTEMI patients was 62 years, 73.9% were male, 83.9% had at least one CV risk factor, 29.5% had prior CVD, and the majority (76.5%) were of Asian ethnicity, as reported previously.^17^

### 3.2 DAPT duration and patient characteristics

Among the 2549 NSTEMI patients, 2315 (90.8%) were discharged on DAPT, of whom 490 (21.2%) stopped DAPT within the first year, and 1832 (79.1%) continued. Among those discharged on DAPT, 2124 (91.7%) survived for at least 12 months thereafter: 299 (14.1%) of these patients stopped within the first year, and 1825 (85.9%) continued. Compared with patients who remained on DAPT for more than 12 months, those who stopped DAPT sooner were older (*P* < .05), less likely to be male (*P* < .01), and were more likely to have diabetes (*P* < .01), a history of CVD (*P* < .05), or chronic renal failure (*P* < .0001) (Table 1).

**Table 1:** Baseline demographic and socioeconomic characteristics of NSTEMI patients who survived ≥12 months and received DAPT for ≤12 months vs >12 months post‐discharge^a^

Characteristic	DAPT duration^a^		*P* value
	≤12 months (n = 299)	>12 months (n = 1825)	
Age group, years			<.05
≤59	117 (39.1)	798 (43.7)	
60‐74	118 (39.5)	748 (41.0)	
≥75	64 (21.4)	279 (15.3)	
Male	205 (68.6)	1382 (75.7)	<.01
BMI, kg/m^2^, mean (SD)	24.4 (3.5)	24.8 (3.7)	.12
Any CVD risk factors^b^	211/297 (71.0)	1300/1818 (71.5)	.87
Hypertension	177/297 (59.6)	1068/1815 (58.8)	.81
Hypercholesterolemia	72/288 (25.0)	362/1765 (20.5)	.08
Diabetes	68/295 (23.1)	548/1799 (30.5)	<.01
Family history of CHD	34/284 (12.0)	172/1702 (10.1)	.34
Current smoker	99/299 (33.1)	616/1825 (33.8)	.93
Obesity	113/292 (38.7)	726/1690 (43.0)	.17
Any previous CVD^b^	102/297 (34.3)	502/1783 (28.2)	<.05
Myocardial infarction	36/297 (12.1)	191/1773 (10.8)	.49
Prior PCI	26/297 (8.8)	150/1781 (8.4)	.85
Prior CABG	4/297 (1.4)	41/1780 (2.3)	.30
Coronary angiogram diagnostic for CAD	37/297 (12.5)	169/1773 (9.5)	.12
Angina	50/297 (16.8)	239/1777 (13.5)	.12
Heart failure	12/294 (4.1)	52/1777 (2.9)	.29
Atrial fibrillation	8/296 (2.7)	32/1776 (1.8)	.30
TIA/stroke	17/296 (5.7)	89/1778 (5.0)	.59
Peripheral vascular disease	3/294 (1.0)	18/1776 (1.0)	.99
Chronic renal failure	21/298 (7.1)	43/1776 (2.4)	<.0001
Any previous/ongoing CV medication^b^	133/288 (46.2)	705/1741 (40.5)	.07
Any antiplatelet medication	81/284 (28.5)	424/1726 (24.6)	.15
Aspirin	74/284 (26.1)	406/1724 (23.6)	.36
Clopidogrel	26/284 (9.2)	146/1723 (8.5)	.70
Beta‐blockers	58/277 (20.9)	279/1662 (16.8)	.09
ACEi/ARB	63/277 (22.7)	329/1661 (19.8)	.26
Statins	58/278 (20.9)	284/1662 (17.1)	.13

*Note*: Values are n (%) unless indicated otherwise.

Abbreviations: ACEi, angiotensin‐converting enzyme inhibitor; ARB, angiotensin receptor blocker; BMI, body mass index; CABG, coronary artery bypass graft; CAD, coronary artery disease; CHD, coronary heart disease; CV, cardiovascular; CVD, cardiovascular disease; IQR, interquartile range; PCI, percutaneous coronary intervention; TIA, transient ischemic attack.

a Includes all patients discharged on two or more antiplatelets (ie, including triple antiplatelet therapy) from the index hospitalization. DAPT duration is defined as the time from discharge to the last use of two or more antiplatelets not accounting for interruptions.

b Number affected/number with data available.

At discharge, DAPT consisted predominantly of aspirin plus a P2Y_12_ receptor inhibitor (99% clopidogrel) (Table 2). After 12 and 24 months of follow‐up, the number of patients on DAPT had declined to 1832 (79.1%) and 1219 (60.0%) patients, respectively. Only 18.4% and 34.9% of patients were on single antiplatelet therapy (SAPT) at 12 and 24 months.

**Table 2:** Antithrombotic management in NSTEMI patients

		Time post‐discharge (months)			
	Discharge	6	12	18	24
	n = 2549	n = 2412	n = 2312	n = 2197	n = 2032
AP therapy, n (%)					
DAPT^a^	2315 (90.8)	2051 (85.0)	1832 (79.1)	1429 (65.0)	1219 (60.0)
Aspirin + P2Y_12_i^b^	2247 (88.2)	2009 (83.3)	1797 (77.7)	1399 (63.7)	1192 (58.7)
Other DAPT	68 (2.7)	42 (1.7)	35 (1.5)	30 (1.4)	27 (1.3)
SAPT	230 (9.0)	328 (13.6)	426 (18.4)	680 (31.0)	710 (34.9)
Aspirin only	111 (4.4)	198 (8.2)	286 (12.4)	538 (24.5)	576 (28.4)
P2Y_12_i^b^	118 (4.6)	128 (5.3)	137 (5.9)	139 (6.3)	130 (6.4)
Other single AP	1 (0.04)	2 (0.1)	3 (0.1)	3 (0.1)	4 (0.2)
No AP therapy	4 (0.2)	33 (1.4)	54 (2.3)	88 (4.0)	103 (5.1)
AC therapy, n (%)	29 (1.1)	23 (1.0)	22 (1.0)	22 (1.0)	20 (1.0)
AC + AP	26 (1.0)	21 (0.9)	20 (0.9)	19 (0.9)	18 (0.9)
AC only	3 (0.1)	2 (0.1)	2 (0.1)	3 (0.1)	2 (0.1)
Death and loss to follow‐up, n					
Death (cumulative)	—	71	127	162	191
Lost to follow‐up (cumulative)	—	66	110	190	326
AP therapy in patients discharged on DAPT^a^ (n = 2315), n (%)	—	n = 2192	n = 2108	n = 2012	n = 1866
DAPT	—	2051 (93.6)	1831 (86.9)	1427 (70.9)	1217 (65.2)
SAPT	—	117 (5.3)	232 (11.0)	506 (25.2)	557 (29.9)
No AP therapy	—	24 (1.1)	45 (2.1)	79 (3.9)	92 (4.9)

Abbreviations: AC, anticoagulant; AP, antiplatelet; DAPT, dual antiplatelet therapy; P2Y_12_i, P2Y_12_ receptor inhibitor; SAPT, single antiplatelet therapy.

a Includes all patients discharged on two or more antiplatelets (ie, including triple antiplatelet therapy) from the index hospitalization. DAPT during follow‐up is defined as the use of two or more antiplatelets not accounting for interruptions.

b 99% clopidogrel.

Among patients who survived ≥12 months post‐discharge, no significant differences were evident in time from symptom onset to admission, or from symptom onset or admission to reperfusion by DAPT duration of use, although patients who stopped DAPT early had a longer median length of hospital stay (9 vs 8 days; *P* < .01) (Table 3). Patients who underwent in‐hospital reperfusion (primary PCI or thrombolysis), in‐hospital PCI/CABG, or drug‐eluting stent (DES) placement were more likely to continue on DAPT beyond 12 months (*P* < .0001 in each case). There were no significant differences in in‐hospital CV complications, degree of dependence at discharge, or EuroQol‐5 Dimensions score between those who stopped DAPT early or continued beyond 12 months.

**Table 3:** In‐hospital and discharge characteristics of NSTEMI patients who survived ≥12 months and received DAPT for ≤12 months vs >12 months post‐discharge^a^

Characteristic	DAPT duration^a^		*P* value
	≤12 months (n = 299)	>12 months (n = 1825)	
Symptom onset to admission, hours; median (IQR)	8.2 (3.0‐22.8)	8.0 (2.8‐20.7)	.98
Admission to reperfusion, hours; median (IQR)	63.1 (18.5‐139.5)	45.6 (7.9‐122.8)	.12
Symptom onset to reperfusion, hours; median (IQR)	72.9 (29.6‐154.5)	59.3 (23.3‐137.8)	.06
Length of hospital stay, days; median (IQR)	9.0 (6.0‐14.0)	8.0 (5.0‐12.0)	<.01
Killip class, n (%)			.51
I	212 (70.9)	1319 (72.3)	
II	55 (18.4)	271 (14.9)	
III	15 (5.0)	108 (5.9)	
IV	6 (2.0)	38 (2.1)	
Missing	11 (3.7)	89 (4.9)	
Left bundle branch block, n (%)^b^	2/286 (0.7)	30/1736 (1.7)	.20
Ejection fraction, n (%)			.76
<30%	5 (1.7)	34 (1.9)	
30‐40%	14 (4.7)	95 (5.2)	
≥40%	196 (65.6)	1137 (62.3)	
Missing	84 (28.1)	559 (30.6)	
Laboratory measures, median (IQR)			
White blood cell count, cells/mm^3^	8170 (6580‐10 385)	8800 (6900‐10 985)	<.05
Initial creatinine, mg/dL	0.94 (0.76‐1.13)	0.91 (0.79‐1.12)	1.00
Glucose, mg/dL	122.0 (100.0‐163.4)	120.0 (99.2‐162.4)	.61
Hemoglobin, g/dL	13.5 (12.2‐14.7)	13.6 (12.3‐14.9)	.47
In‐hospital reperfusion,^b^^,^ c n (%)	143 (48.0)	1246/1805 (69.0)	<.0001
In‐hospital PCI/CABG,^b^ n (%)	145 (48.7)	1253/1806 (69.4)	<.0001
Number of dilated vessels, median (IQR)	1.0 (1.0‐2.0)	1.0 (1.0–2.0)	.85
Any DES^b^ n (%)	127 (42.5)	1113/1825 (61.0)	<.0001
In‐hospital complications,^d^ n (%)^b^	44 (14.9)	216/1815 (11.9)	.14
*Discharge characteristics*			
Anticoagulants at discharge, n (%)	2 (0.7)	12 (0.7)	.98
Dependence at discharge, n (%)			.41
No dependence	282 (94.3)	1681 (92.1)	
Nonsevere dependence	16 (5.4)	134 (7.3)	
Severe dependence	1 (0.3)	10 (0.6)	
EQ‐5D overall score, mean (SD)	77.4 (13.9)	76.8 (15.1)	.48
EQ‐5D simple score, n (%)^b^			.74
0	185/298 (62.1)	1090/1824 (59.8)	
1	38/298 (12.8)	254/1824 (13.9)	
≥2	75/298 (25.2)	480/1824 (26.3)	

*Note*: Values are n (%) unless indicated otherwise.

Abbreviations: CABG, coronary artery bypass graft; DAPT, dual antiplatelet therapy; DES, drug‐eluting stent; EQ‐5D, EuroQol‐5 Dimensions quality of life questionnaire; IQR, interquartile range; MI, myocardial infarction; PCI, percutaneous coronary intervention.

a Includes all patients discharged on two or more antiplatelets (ie, including triple antiplatelet therapy) from the index hospitalization. DAPT duration is defined as the time from discharge to the last use of two or more antiplatelets not accounting for interruptions.

b Number affected/number with data available.

c Primary PCI or thrombolysis.

d Included MI, recurrent ischemia, heart failure, cardiogenic shock, and arrhythmia.

### 3.3 DAPT duration and outcomes

**Figure 1:**
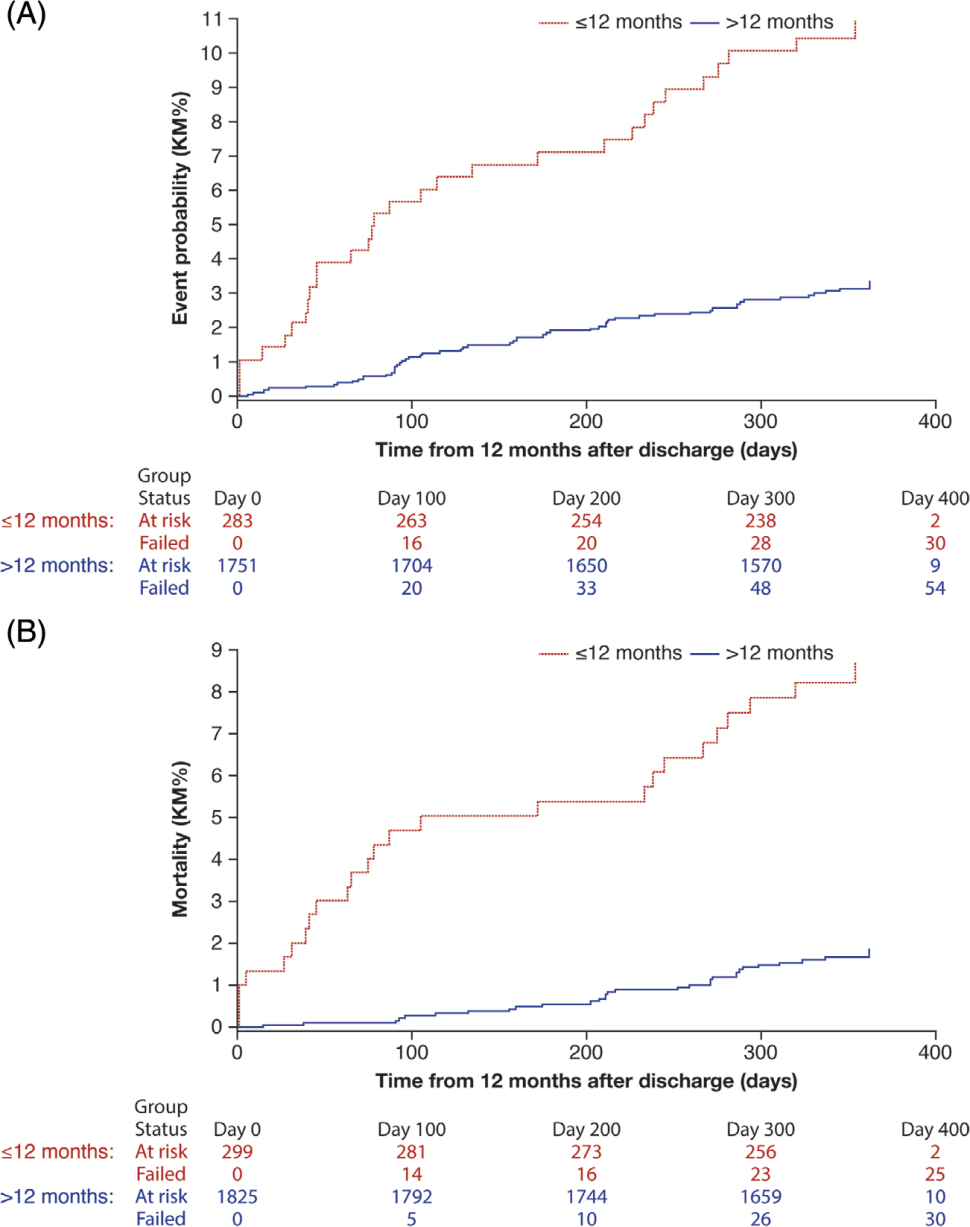
Cumulative incidence of, A, the composite endpoint and, B, mortality over the following 12 months in NSTEMI patients who survived ≥12 months and received DAPT for ≤12 months or >12 months post‐discharge.*The incidence of the composite endpoint, death, MI, and stroke, over the second 12 months was 10.6% with DAPT ≤12 months and 3.1% with DAPT >12 months, and the mortality rate was 8.4% and 1.6%, respectively

In the NSTEMI population who survived at least 12 months post‐discharge, the median (interquartile range) duration of DAPT in patients who received it for ≤12 months and > 12 months was 219 (80‐347) and 718 (589‐725) days, respectively. The incidence of the composite endpoint, death, MI, and stroke, over the next 12 months was 10.6% with DAPT ≤12 months and 3.1% with DAPT >12 months, and the mortality rate was 8.4% and 1.6%, respectively (Table 4 and Figure 1). There were only four patients with major bleeding, all in the DAPT >12 months group.

**Table 4:** Event rates over the following 12 months in NSTEMI patients who survived ≥12 months, and received DAPT for ≤12 months or > 12 months post‐discharge

Event	Event rate by DAPT duration, n/N (%)	
	≤12 months (n = 299)	>12 months (n = 1825)
Composite of death, MI, and stroke	30/283 (10.6)	54 (1751 (3.1)
Composite of death, MI, stroke, and major bleeding	29/281 (10.3)	58/1750 (3.3)
Mortality	25/299 (8.4)	30/1825 (1.6)
Myocardial infarction	6/273 (2.2)	21/1766 (1.2)
Stroke	4/279 (1.4)	11/1810 (0.6)
Major bleeding	0	4/1824 (0.2)

Abbreviations: n/N, number affected/number with data available.

## 4 DISCUSSION

The results of this analysis of NSTEMI patients included in the EPICOR Asia study showed that the majority (90.8%) were discharged on DAPT, 78.8% continued it for over 12 months, and 60.0% remained on DAPT at the final 2‐year follow‐up visit. Patients who discontinued DAPT within the first year appeared to be a higher‐risk population, as they were older, and more likely to have a history of CVD or renal dysfunction. Conversely, patients treated with DAPT beyond 1 year appeared to be at lower CV risk. This reflects the observed paradox that higher‐risk patients post‐MI are less likely to persist with prescribed medication.^18^ A recent Canadian observational study in 2034 acute MI patients (NSTEMI or STEMI) who had undergone PCI also reported higher baseline risk among patients who discontinued DAPT early compared with at least 12 months of treatment.^19^ In that study, significant differences in baseline characteristics with shorter vs longer DAPT duration included older age, history of MI, heart failure, or atrial fibrillation, and a higher Global Registry of Acute Coronary Events (GRACE) score (all *P* < .001 for trend). The authors also reported a higher incidence of a composite primary outcome (nonfatal acute MI, unplanned coronary revascularization, stent thrombosis, new or worsening heart failure, cardiogenic shock, or stroke) in patients who discontinued DAPT in less than 6 weeks (*P* < .0001), and 6 weeks to <6 months (*P* = .02), but not for 6 months to <12 months (*P* = .06) compared with DAPT ≥12 months, indicating that the highest risk of events is in the earliest stages following an acute MI. This appears to be reflected in the EPICOR Asia data, since most of the significant differences in baseline characteristics between the shorter vs longer DAPT duration populations either diminished or disappeared when only the ≥12‐month survivors were included in the analysis, with the exception of male sex and chronic renal failure.

Recent observational studies have also examined DAPT duration in NSTEACS patients in Asia, but their follow‐up periods typically did not exceed 1 year.^20^, ^21^ For example, the Taiwan ACute CORonary syndrome Descriptive registry (T‐ACCORD), performed in 2004 to 2007, included 1331 survivors of hospitalization for NSTE‐ACS (32% NSTEMI and 68% UA).^20^ Here, the investigators reported that 62% of patients were discharged on DAPT, but only 13% remained on DAPT at 12 months, indicating weak implementation of treatment guidelines. Notably, however, persistence with DAPT for ≥9 months was associated with a significantly lower 1‐year cumulative mortality compared with DAPT use for <9 months (*P* < .01 by log rank test).

Low adherence to guideline‐based DAPT use was demonstrated in a further study in Malaysia of 525 high‐risk survivors of NSTE‐ACS (Malaysia‐ACute CORonary syndromes Descriptive [ACCORD] study). Here, more than 80% of patients were managed medically following hospitalization with 48% discharged on DAPT; this declined to 32% and 16% at 3 and 12 months, respectively.^21^ Although the authors did not report on CV outcomes, this was a younger population than in EPICOR Asia and a high‐risk population with only 3% having no identifiable CV risk factor.

In fact, the high‐risk population should be given longer duration of DAPT than others, which was verified in THEMIS (The Effect of Ticagrelor on Health Outcomes in diabetes Mellitus patients Intervention Study).^22^ The results showed that patients aged 50 years or older with stable CAD and diabetes, even without a history of MI or stroke, had a lower incidence of ischemic CV events with long‐term DAPT (median duration 39.9 months) vs single aspirin therapy. The THEMIS‐PCI substudy showed that in the prespecified subgroup of patients with diabetes, stable CAD, and previous PCI, long‐term DAPT (median duration 3.3 years) provided a favorable net clinical benefit, more so than in patients without a history of PCI.^23^

In EPICOR Asia, the “real‐world” trial, the incidence of major adverse CV events, including mortality, during the second year of follow‐up among patients who survived at least the first year was higher with ≤12 months of DAPT compared with >12 months of DAPT. Furthermore, the higher incidence of the composite endpoint in patients who received DAPT for up to 12 months appeared largely driven by cumulative mortality. As stated earlier, however, a causal relationship cannot be inferred between earlier DAPT discontinuation and increased risk of subsequent events. In the “real‐world” setting, the reasons contributing to DAPT use and duration will be multifarious. It is possible that at least some cases of early DAPT discontinuation are driven by bleeding events, and patients at increased risk of bleeding are also often at increased risk of ischemic events, and vice versa.^24^ The results of other studies referred to above also suggest that ACS patients who stop DAPT earlier, for any reason, are a potentially high‐risk group requiring careful monitoring and management.

### 4.1 Limitations

Limitations of the present analysis include those usually applicable to observational studies, such as the nonrandomized nature of the study population. There is also the risk of immortal time bias; that is, bias introduced by the study design in which an outcome cannot occur during the specified period of follow‐up.^25^ In this study, for example, patients had to survive until the 12‐month follow‐up visit in order to be included in the prolonged DAPT group, necessarily inferring an advantage for these patients. Such potential bias may be evident in the somewhat counter‐intuitive observation that NSTEMI patients with a prior history of CVD in EPICOR Asia were more likely to discontinue DAPT early. This could be explained by the possibility that their overall health was worse and they were more likely to have died during the first 12 months and therefore had no opportunity to continue on DAPT for >12 months. Our analysis of baseline risk factors did show that many of the significant differences between shorter vs longer overall DAPT duration populations became less or nonsignificant when we focused on patients who survived the first 12 months. It should also be noted that most patients were enrolled at well‐equipped centers, perhaps fostering greater uptake of evidence‐based guidelines, specifically, the use of DAPT.

Finally, while the reasons for DAPT discontinuation were not reported, some may be suggested. For example, baseline data showed that patients who were older, female, with low body mass index (BMI), previous CVD, or renal insufficiency tended to stop DAPT treatment soon after discharge. The early cessation of DAPT in the older, low‐BMI patients may be associated with bleeding, such as epistaxis and gingival bleeding. Although such bleeding is usually mild, the patient may be very nervous and likely to stop DAPT as soon as possible. Early discontinuation of drugs in women may be related to lack of awareness of ACS disease, low family and social status, and insufficient attention. In patients with previous CVD or renal insufficiency, early withdrawal of medication may be associated with too many concomitant drugs or treatment with DAPT for a period prior to study enrollment. Furthermore, in some cases, taking too much medicine can cause stomach discomfort or ulcers. Sometimes, for example, aspirin therapy may be stopped, either under the guidance of a doctor or by the patient him/herself. As premature cessation of DAPT can increase the risk of CV events, additional attention should be paid to these groups, including patient education, increased follow‐up frequency, and close guidance on antiplatelet therapy and other treatments.

## 5 CONCLUSIONS

In summary, the majority of NSTEMI patients in EPICOR Asia were discharged on DAPT, and nearly two‐thirds remained on it at 2 years. NSTEMI patients in whom DAPT treatment is stopped earlier than 12 months post‐discharge appear to represent a vulnerable group in terms of baseline risk factors and may benefit from more intensive monitoring during long‐term follow‐up.

## DISCLOSURES

Yanan Zou, Shuang Yang, Shipeng Wang, Bo Lv, Lili Xiu, Lulu Li., Stephen W.‐L. Lee, Yong Huo, and Bo Yu have nothing to disclose. Chee Tang Chin has received research support from Eli Lilly; honoraria from Medtronic, and has been a consultant or advisory board member for AstraZeneca. Stuart J. Pocock receives research funds from AstraZeneca.
